# Bowel cancer screening in England: a qualitative study of GPs' attitudes and information needs

**DOI:** 10.1186/1471-2296-7-53

**Published:** 2006-09-18

**Authors:** Chris Woodrow, Linda Rozmovits, Paul Hewitson, Peter Rose, Joan Austoker, Eila Watson

**Affiliations:** 1 CRUK-Primary Care Education Research Group, Department of Primary Care, University of Oxford, Oxford, UK

## Abstract

**Background:**

The National Health Service Bowel Cancer Screening Programme is to be introduced in England during 2006. General Practitioners are a potentially important point of contact for participants throughout the screening process. The aims of the study were to examine GPs' attitudes and information needs with regard to bowel cancer screening, with a view to developing an information pack for primary care teams that will be circulated prior to the introduction of the programme.

**Methods:**

32 GPs participated in semi-structured telephone interviews. 18 of these had participated in the English Bowel Screening Pilot, and 14 had not. Interviews covered attitudes towards the introduction of the Bowel Cancer Screening Programme, expected or actual increases in workload, confidence in promoting informed choice, and preferences for receiving information about the programme.

**Results:**

GPs in the study were generally positive about the introduction of the Bowel Cancer Screening Programme. A number of concerns were identified by GPs who had not taken part in the pilot programme, particularly relating to patient welfare, patient participation, and increased workload. GPs who had taken part in the pilot reported holding similar concerns prior to their involvement. However, in many cases these concerns were not confirmed through GPs experiences with the pilot. A number of specific information needs were identified by GPs to enable them to provide a supportive role to participants in the programme.

**Conclusion:**

The study has found considerable GP support for the introduction of the new Bowel Cancer Screening Programme. Nonetheless, GPs hold some significant reservations regarding the programme. It is important that the information needs of GPs and other members of the primary care team are addressed prior to the roll-out of the programme so they are equipped to promote informed choice and provide support to patients who consult them with queries regarding screening.

## Background

Colorectal cancer is the second most common cause of cancer death in the UK [1]. Evidence from randomized controlled trials has confirmed that screening for colorectal cancer using Faecal Occult Blood testing (FOBt) as the primary screening modality can significantly reduce colorectal cancer mortality [2]. Two pilot screening rounds, which commenced in 2000, were implemented in order to assess the viability of a national screening programme for bowel cancer being undertaken within the UK National Health Service. Data from the first pilot round demonstrated that FOBt screening for colorectal cancer is feasible within the context of the NHS [3]. A national programme of screening is expected to be rolled out in 2006.

The National Health Service Bowel Cancer Screening Programme (BCSP) will be centrally organised in England, with call-recall services and testing and analysis of FOBt kits performed by five central screening hubs across the country. If a participant receives a positive FOBt result they will be offered an appointment with a specialist nurse to discuss the implications of this at a local screening centre. GPs will also be notified of the participant's positive FOBt result. Primary care will not have direct responsibility for the recruitment or delivery of the programme. Therefore, their involvement is likely to be concerned with administrative duties (such as dealing with letters and reports generated by the screening units) and providing information to patients who have queries about the screening process [4].

GPs may be an important source of information for patients who have queries about bowel cancer screening. In healthcare systems outside the UK (where primary care is more directly involved with the screening process), GPs have been identified as the preferred source of information about bowel cancer [5] and the person most influential in patient's decisions regarding testing [6], with GP motivation associated with uptake of screening [7]. If GPs are to effectively provide information to patients with queries about the screening programme, it is important to explore their attitudes towards the BCSP. Informed choice is a central strand to the BCSP, with the National Screening Committee advising that both the risks and benefits are made explicit to individuals invited to cancer screening [8]. GPs could play a role in the facilitation of informed choice in patients who consult them about participating in bowel cancer screening, a task entirely dependant on the provision of relevant, accurate and complete information. Therefore, it is also important to understand the specific information needs of general practitioners involved with bowel cancer screening.

This report describes the findings from qualitative interviews conducted with GPs who either had, or had not been involved in the English Bowel Screening Pilot. The aims of the study were to examine GPs' attitudes and information needs with regard to bowel cancer screening, with a view to developing an information pack for primary care teams that will be circulated prior to the introduction of the programme.

## Methods

Semi-structured telephone interviews with 32 General Practitioners (GPs) were conducted between April and June 2005. The sample consisted of both GPs practising in areas participating in the pilot phase of the BCSP, and GPs practising outside these areas. This allowed the comparison of attitudes and information needs between individuals with differing levels of knowledge and experience of the screening programme. Letters of invitation were sent to 297 GPs from 93 practices in areas participating in the pilot phase of the BCSP. Convenience sampling was used to recruit GPs practising outside these areas. GPs were remunerated £50 for participation.

Eighteen GPs (13 males and 5 females) were recruited from 15 practices participating in the pilot phase of the BCSP. These participants were based in practices located in the Coventry, Rugby, and North Warwickshire areas of England, covering locations ranging from rural to inner city. The majority of participants described their populations as lower middle to middle class, with a minority indicating that their practices covered areas of severe deprivation. The majority of GPs indicated that fewer than 10% of their practice populations were from ethnic minority backgrounds, with three indicating that they had large Asian populations.

The remaining fourteen GPs (6 males and 8 females) were recruited from practices in areas that did not participate in the pilot phase of the BCSP. These individuals were based in various locations in England, ranging from rural to inner city. Most participants indicated that their patient populations were socio-economically mixed, and a small number indicated that they worked in areas of severe deprivation or relatively affluent areas. Five GPs indicated that they had substantial or increasing numbers of ethnic minority patients, largely Asian.

Telephone interviews were conducted by LR, an experienced interviewer. Interviews were tape recorded with the participant's consent and lasted approximately 20 – 30 minutes. Two semi-structured interview schedules developed by LR and EW were used for this purpose, one for use with pilot GPs and one for use with the general population GPs. These covered participant's attitudes towards the introduction of the national bowel cancer screening programme, any expected or actual increases in workload, their confidence in promoting informed choice, and their preferences for receiving information about the programme. Interviews were conducted with both groups until data saturation was reached.

Interviews were transcribed verbatim, with the accuracy of each recording verified by the interviewer. A coding frame was constructed and applied using the HyperResearch software package by LR. Interviews were analysed for anticipated and emergent themes using a grounded theory approach [9]. The analysis included searches for disconfirming evidence. Coding and interpretation of the data was regularly discussed with EW.

## Results

The overall findings are described below in terms of the main themes that emerged. After each quote is a descriptor indicating whether or not the GP had participated in the pilot phase of the BCSP, together with an individual research number.

### Attitudes

Strong support for the introduction of a national screening programme for bowel cancer was shown by many of the pilot and non-pilot GPs in this study. However, whilst welcoming the screening programme, a number of GPs from both groups expressed certain reservations. Reservations held by Non-pilot GPs fell into three broad themes. These related to the welfare of participating individuals, levels of patient participation and increased workload for primary care. Pilot GPs reported holding similar reservations prior to their involvement in the programme, although many reported that during the course of the pilot these did not materialise.

### Positive attitudes

The benefit of early detection, the non invasive nature of FOB testing, and knowledge of research evidence demonstrating mortality reductions by FOB testing were cited by pilot and non-pilot GPs as reasons for their positive attitudes towards the introduction of the BCSP.

"*... The basic research underlying it was done years ago ... and there was absolutely no doubt that it would save lives ... So ... I'm very positive about it. I think it's long overdue." (NON-PILOT 11)*

"*... this is the sort of thing we should be doing ... if there's a reasonable chance that we can improve detection and treatment of colorectal cancer then you know it's worth doing ... "(PILOT 3)*

In addition to this, many of the pilot GPs commented, unprompted, on how well the pilot programme had been run:

"*I haven't really had any problems with this pilot at all. I mean it's been a remarkably smooth introduction" (PCA2)*

### Reservations

#### Patient welfare

An issue of concern to a considerable number of non-pilot GPs was the impact of an increasing culture of screening on people's lives. Some felt concerned about screening saturation and the increasing number of interventions that patients were subjected to:

"*... you're sometimes concerned that we're getting to a state where we're screening this and screening that ..." (NON-PILOT 13)*

The generation of patient anxiety was a concern for many pilot and non-pilot GPs. Several GPs felt that the possibility of detecting an abnormality must be weighed off against potential anxiety generated by the screening process:

"*... we're already creating a country of worried well and ... we will add to that because some of the early signs of bowel cancer are so common ... They'd have to convince me that the faecal occult test was so good at picking up silent malignancies that it was worth worrying the life out of the majority of our patients." (NON-PILOT 12)*

A related concern was that of the possibility of patients taking up FOBt screening without fully realizing the potential consequences, and dropping out of the process when offered a colonoscopy:

"*I mean if they don't want that [a colonoscopy] then they shouldn't start, that's the thing. They should live in ignorance and not start ... and then find that they can't complete it." (NON-PILOT 10)*.

The possibility of specific groups of participants being particularly susceptible to anxiety generated by the screening programme was also raised. Two non-pilot GPs in particular felt that the introduction of a new screening programme had the potential to generate serious anxiety within their communities. One of these served a large ethnic minority population which had little previous experience of screening, whilst the second was a GP working with mainly poorly educated manual workers in a community considered to have unusually high levels of pathology.

Issues relating to the reliability of the FOB test were also raised by pilot and non-pilot GPs alike. Of particular concern were anxiety caused by false positive results, false reassurance derived from normal FOB test results, and patients being subjected to (sometimes unwarranted) invasive procedures through the screening programme.

"*I need convincing that the faecal occult test is not going to produce a lot of false positives that will engender a lot of worry in people ... I also want to be convinced that we're not going to then engender a false sense of security ... they start getting firm symptoms, change of bowel habit, 'But I've had the test done and it was normal so I won't go."' (NON-PILOT 12)*.

Some individuals suggested that the possible risks of screening ought to be made more apparent to potential participants:

"*....patients just see the early pick-up as the end goal ... I think the false positive, false negative bit of it often passes people by." (NON-PILOT 5)*

Many GPs raised concerns that managing the patient anxiety generated by false positives was likely to result in additional responsibility for GPs, although felt that this was an inevitable and acceptable part of any screening programme.

However, whilst around half of pilot GPs were expecting an increase in consultations with individuals worried by test results or other aspects of the programme, this did not seem to be a major issue in practice:

"*Well we thought that ... we'd be much more swamped with false positive patients who'd be frightened and it just didn't happen." (PILOT 14)*

#### Patient participation

Concerns regarding patient participation in relation to the screening programme were raised by several non-pilot GPs. Patient's reluctance to address issues relating to their bowels was a commonly cited barrier to participation by GPs:

"*I think you're much more likely to get people querying whether they want to take part in it or not ... because I think people don't want their bowels looked into thank you very much. I think you very much get the impression from patients, they'd almost rather not know ... So I personally think the uptake's going to be lower and I think that might be a problem for the screening programme." (NON-PILOT 1)*

Furthermore, patients who tended not to be proactive about their health were felt to be a cause for concern, since they may not participate in screening. Several GPs felt that some patients may be disadvantaged because of their age, incapacity, level of education or English language proficiency:

"*... the worry I have is about motivation of the patients to do it. I mean they're all elderly, some of them will have other disabilities, they may find it difficult to understand the instructions, difficult to see the point of it, and I feel that ... with the sort of patients we have ... people who can't read English or people who aren't very well educated, they're the ones that are going to have a higher incidence of bowel cancer and they're the ones who are going to fail to comply ... and not get it done." (NON-PILOT 11)*

Similarly, several pilot GPs said they had anticipated problems with patient participation prior to the pilot. However, many GPs ultimately reported that during the course of the pilot they received no indication of low rates of participation:

"*I've not had anyone ... actually come and say 'I don't know what to do', 'I don't know how to do it', I can't do it, I don't want to do it' and so on. So I presume that they've either done it or they've not done it and not told me." (PILOT 12)*

One Pilot GP practising in an area of urban deprivation with a high proportion of ethnic minority (Asian) patients found that the response rate exceeded expectations:

"*... at the beginning I thought they won't be responding because when you try to screen for cervical cytology among the females ... the response is not very good ... So ... I felt that the response was pretty good actually by my standards of the practice." (PILOT 13)*

#### Additional Workload

Most non-pilot GPs envisaged that the introduction of screening would result in an increase in primary care workload, with an increase in patient consultations due to worried individuals seeking information about screening of concern:

"*... the increased workload I think would come largely from people wanting information or wanting to discuss their results or wanting reassurance or explanation about the test ... if something like this is rolled out nationally they will want to come and talk about it" (NON-PILOT 2)*

The quality of patient information provided by the screening programme was, therefore, considered to be a key factor in regulating this likely increase in primary care workload:

"*... it depends partly on the quality of ... information that's given to the public ... I think that's very important, the information that goes out to the actual people when they're sent their letters to take part in the screening and how much the impact on us, you know ... If that's not clear enough then it will be their first port of call in terms of queries." (NON-PILOT 1)*

Similarly, ensuring that the information provided to primary care teams would allow both nurses and receptionists to field patient enquiries was considered to be important in absorbing the anticipated workload:

"*... the practice nurses often, you know, people feel most easy about approaching them with questions about that sort of thing ... I think everybody will need to be informed and able to answer questions, and you know leaflets and resources around reception and places like that." (NON-PILOT 5)*.

However, none of the non-pilot GPs saw the potential increase in workload as a serious problem, describing this as an appropriate use of their time. Many expected that the additional workload would decrease over time as screening becomes more established, and the public more aware of the programme. Others felt that additional workload in the short term would be reduced in the future if screening leads to earlier diagnosis of bowel cancer and better polyp surveillance:

Most pilot GPs, like the non-pilot GPs, had anticipated an increase in workload in terms of increased patient appointments. Pilot GPs anticipated patient queries from either patients requiring assistance with completing the test, or patients who were worried about the screening process or their results. However, only a small minority of individuals reported seeing any actual increase in patient numbers, and GPs reported that queries were usually included in appointments booked with regard to other matters. Pilot GPs also considered this to be an appropriate use of their time.

Although patient information leaflets were available in a variety of languages during the pilot, two GPs whose practices covered large ethnic minority populations did notice increase in patient numbers:

"*Well I mean there was a fair amount of work involved because patients would bring all these forms to us and say 'what's this?' because you know a lot of our Asian patients don't speak English ... So there was more work for us. But I mean I still think it was worthwhile doing ... in some ways it was a good thing because we could then impress upon them the importance of doing it ..." (PILOT 17)*.

Pilot GPs reported that patients requiring information or input were usually seeking endorsement of the screening programme, clarification of how to use the kit, reassurance after a positive FOBt, further details about colonoscopy, or explanations in languages other than English.

### Informed choice and Information needs

Whilst many of the non-pilot GPs held generally positive attitudes with regard to the screening programme, only a few individuals indicated that they currently felt able to promote informed choice in patients who consulted them about taking part in screening. The majority said that they would need additional information in order to be able to achieve this. A description of the entire screening pathway was commonly cited as a requirement by GPs. Other areas of information needed to promote informed choice identified by non-pilot GPs were evidence of the risks and benefits of screening, technical data about the sensitivity and specificity of FOB testing and evidence of detection and survival rates.

"*I don't have the information at my fingertips about the risk and benefits ... properly and clearly explained and that's one of the hardest things, I think, ... in actual clear language." (NON-PILOT 1)*

Most pilot and non-pilot GPs expressed the desire for accessible statistical information about the screening programme to be made available to them in order to fully inform patients. The use of graphics and charts were cited as useful inclusions, particularly since these made it easier to explain complex information to individuals who may have difficulty understanding verbal information, or who do not have good English language skills. Similarly, access to all the information sent to patients by the screening programme was seen as essential. Many GPs agreed that future information packs containing both quick reference elements and more detailed, clinician orientated information would best serve the needs of GPs in the provision of information. GPs expressed the view that quick reference information would be the most useful item for many GPs.

Many of the pilot GPs had little recollection of the information pack provided to them during the course of the pilot, and indicated that they had used it only rarely. Rather than any inadequacy with the information, however, this was largely attributed to the efficiency of the screening pilot and the briefing sessions GPs had attended:

"*...my recollection is simply that we were primed by face to face meetings in the first place and therefore were aware, and I don't necessarily think that the information pack really added much to that. It was useful as a reference tool, but I mean I don't think I've even had to refer to it at all." (PILOT 12)*

While fully endorsing informed choice as a concept, a minority of the non-pilot GPs questioned the reality of promoting it in practice:

"*... I think it's absolutely right that we should say that patients have informed choice but the reality is that they ... would come to me and say, 'What shall I do doctor?' and they want an answer from me personally ... quite a lot of patients actually don't want the information. They can't cope with it. They don't know what to do with it. Therefore they're looking at you as a person to sort of say 'What shall I do?' And we then, even though we may not want this role, have to sort of advise them, just simplistically." (NON-PILOT 9)*

"*...we talk about informed choice all the time, but in every instance we're pushing people to accept essentially the national guidelines ... as with PSA testing, they're making an informed choice. But nevertheless we're trying to get them to choose not to have it done in most cases. And with cervical smears as well it's an informed choice, but really is there any choice you know? We're pushing them very very hard to have it done. So the emphasis is on the informed, not the choice." (NON-PILOT 11)*

## Discussion

The GPs in this study were generally positive towards the introduction of the BCSP, regardless of their involvement in the pilot phase of screening. However, GPs held a number of reservations with regard to the programme. Prominent amongst these were concerns for the welfare of potential screening participants, relating to the impact of an increasing culture of screening on people's lives, the potential anxiety caused by the screening process, and issues with the reliability of the FOB test which may impact on patients. The need for good information materials to support the whole primary care team was emphasized, as was the importance of quality patient information. GPs also expressed concerns relating to levels of patient participation in the programme, and the additional workload which the new programme may generate for primary care. Both pilot and non-pilot GPs held similar concerns with regard to the introduction of the BCSP. However, these concerns were rarely confirmed through the experience of the pilot GPs.

The study provides a unique qualitative examination of the attitudes of GPs towards the first new cancer screening programme to be introduced in England for several years. The sample included both GPs who have been involved in the bowel cancer screening pilot and those who have not. GPs practiced in a range of locations, and with diverse patient populations. A possible limitation of the study is the small number of GPs involved, although the vast majority of GPs came from different practices. Whilst data saturation was reached, a convenience sample was used to recruit non-pilot GPs which may have been biased towards individuals holding positive attitudes regarding screening for bowel cancer. Furthermore, only a small number of GPs in the sample reported practising in areas with large numbers of ethnic minority patients. Ethnic minority groups were associated with lower levels of participation in the pilot [10], and those GPs in the current sample who did practice in areas with large numbers of these participants reported increases in patient appointments. Therefore, further work with GPs such as these, who may have different experiences with the programme, is required.

The concerns raised by GPs relating to the welfare of screening participants are not unique to bowel cancer screening. UK GPs are generally positive about breast screening, for example, although hold concerns relating to pain and discomfort caused by testing, false reassurance derived from results, and problems caused by false positive results [11]. Evidence from outside the UK healthcare system has also highlighted GPs' concerns with screening for Bowel Cancer (although primary care involvement in screening differs in countries outside the UK). Family Doctors in one Canadian study reported rarely using FOB testing for screening due to a number of serious limitations, including low yield, high rates of false positives and false negatives, and a number of technical problems [12]. Similarly, only 38% of GPs in an Australian survey study indicated the belief that FOB testing was an effective means of reducing premature death from bowel cancer [13].

The GPs in this study highlighted the importance of the provision of high quality information to all parties involved in the National Health Service Bowel Cancer Screening Programme – both health professionals and patients. The significance of providing evidence based information to support GPs and promote shared decision making in bowel cancer screening has been highlighted elsewhere [12,13], and in the UK, primary care teams are provided with detailed information to assist in providing information to patients on the risks and benefits of the PSA test as part of the Prostate Cancer Risk Management Programme [14]. The provision of high quality information to patients is similarly important in decision making regarding screening, and decision aids can increase knowledge and reduce decisional conflict in individuals deciding whether or not to participate in screening [15]. Informed choice is a central theme of the new screening programme [8], and whilst GPs in the current study indicated that they would be willing to promote informed choice amongst potential screening participants, they stressed that their own information needs must first be addressed. It is important that information materials for primary care are matched to GPs information preferences. The most important factor for many GPs related to ease of use on a day to day basis. Concise information that can be quickly digested by GPs, understood by patients, and used by all members of the primary care team would maximise the efficiency of information provision to potential screening participants. Similarly, whilst GPs accepted that managing patient anxiety is an acceptable use of their time, a number felt that the risks of screening ought to be made more apparent to potential participants at the outset. GPs felt that the provision of high quality information to patients would promote understanding of the risks and benefits of screening, and may therefore help to alleviate some of the potential problems experienced by patients. This in turn could alleviate some of the additional workload impact on the GPs themselves.

GPs and other members of the primary care team will not be directly involved in organisational aspects of the BCSP, although they are a potentially important point of contact for patients with queries relating to any stage of the screening process. Pilot GPs in this study did not report large numbers of additional appointments, reflecting the "modest but discernable" increase reported in a previous survey and audit of practices participating in the pilot [4]. However, survey data from the first round of the screening pilot indicated that a significant proportion of individuals with positive test results and screen-detected cancers consulted their GPs [10], and it is inevitable that there will be some increase in patient queries during the roll out of the programme. Therefore, the support of primary care will be important for the new programme. It is interesting to note that whilst pilot GPs reported rarely meeting worried patients and surprise at the apparent high level of participation, data from the first round of the screening pilot indicates that overall uptake was 59% in England, with uptake lowest in areas with the highest proportion of residents from the Indian-subcontinent [10]. Indeed, the GPs in the current sample with significant numbers of ethnic minority patients proved to be an exception in that they did experience significant increases in patient consultations, reporting that they were happy to be in a position to assist these individuals. When the national programme commences, primary care may be well placed to assist in providing information and support to hard to reach groups with special needs, such as those with poor English language skills, complications caused by pre existing medical conditions, or unusually high levels of anxiety.

It is also important to note that pilot enjoyed particularly high standards of efficiency, which compelled a number of GPs to comment unprompted on how well it had been run. The pilot GPs in our study rarely reported using the educational materials provided to them. The main reason cited for this was the exceptional quality of briefing sessions, and the high level of support provided both to primary care and to patients by the screening team. If this quality is not maintained, therefore, then the provision of high quality information will be of greater significance both to primary care and to patients during the programme itself than during the pilot.

## Conclusion

In conclusion, this study has found considerable GP support for the introduction of the new Bowel Cancer Screening Programme. Nonetheless, GPs hold some significant reservations about the programme. Education prior to the start of the programme and regular feed-back once the programme is up and running are essential to ensure primary care support. The findings from this study have informed the development of an information pack for primary care teams that will be circulated prior to the introduction of the programme.

## Competing interests

The author(s) declare that they have no competing interests.

## Authors' contributions

CW drafted the manuscript.

LR conducted interviews and data analysis.

PH assisted in drafting the manuscript.

PR participated in the study design and assisted in recruitment of study participants.

JA assisted in drafting the manuscript.

EW participated in the study design, and assisted in data analysis drafting the manuscript

All authors read and approved the final manuscript.

## Pre-publication history

The pre-publication history for this paper can be accessed here:


